# Fitness Advantage of *mcr-1*–Bearing IncI2 and IncX4 Plasmids *in Vitro*

**DOI:** 10.3389/fmicb.2018.00331

**Published:** 2018-02-27

**Authors:** Renjie Wu, Ling-xian Yi, Lin-feng Yu, Jing Wang, Yiyun Liu, Xiaojie Chen, Luchao Lv, Jun Yang, Jian-Hua Liu

**Affiliations:** Guangdong Provincial Key Laboratory of Veterinary Pharmaceutics Development and Safety Evaluation, College of Veterinary Medicine, South China Agricultural University, Guangzhou, China

**Keywords:** *Escherichia coli*, *mcr-1*, colistin, plasmid, fitness

## Abstract

The objective of this study was to assess the impact of diverse plasmids bearing colistin resistance gene *mcr-1* on host fitness. Forty-seven commensal *E. coli* isolates recovered from the pig farm where *mcr-1* was first identified were screened for *mcr-1. mcr-1*-bearing plasmids were characterized by sequencing. The fitness impact of *mcr-1*-bearing plasmids was evaluated by *in vitro* competition assays. Twenty-seven (57.5%) *E. coli* isolates were positive for *mcr-1*. The *mcr-1* genes were mainly located on plasmids belonging to IncI2 (*n* = 5), IncX4 (*n* = 11), IncHI2/ST3 (*n* = 8), IncFII (*n* = 2), and IncY (*n* = 2). InHI2 plasmids also carried other resistance genes (*floR, bla*_CTX−M_, and *fosA3*) and were only detected in isolates from nursery pigs. Sequences of the representative *mcr-1*–bearing plasmids were almost identical to those of the corresponding plasmid types reported previously. An increase in the fitness of IncI2- and IncX4-carrying strains was observed, while the presence of IncHI2, IncFII and IncY plasmids showed a fitness cost although an insignificant fitness increase was initially observed in IncFII or IncY plasmids-containing strains. Acquisition of IncI2-type plasmid was more beneficial for host *E. coli* DH5α than either IncHI2 or IncX4 plasmid, while transformants with IncHI2-type plasmid presented a competitive disadvantage against IncI2 or IncX4 plasmid containing strains. In conclusion, IncI2, IncX4, and IncHI2 were the major plasmid types driving the dissemination of *mcr-1* in this farm. Increased fitness or co-selection by other antimicrobials might contribute to the further dissemination of the three epidemic *mcr-1*–positive plasmids (IncI2, IncX4, and IncHI2) in this farm and worldwide.

## Introduction

The ongoing emergence and spread of multidrug-resistant and even pan drug-resistant Enterobacteriaceae has led the world into a post-antibiotic era (Bush et al., 2011; Laxminarayan et al., 2013). Under these circumstances, colistin, as the last resort for the treatment of infections with multidrug-resistant (MDR) bacteria, has gained extensive attention. However, colistin resistance has been found in gram-negative bacteria. Most importantly, transferable colistin resistance, which was first found to be mediated by *mcr-1*, emerged in 2015 (Liu et al., 2016). Since then, research groups have increasingly screened for the presence of *mcr-1*, and this gene has thus far been detected in diverse Enterobacteriaceae species (*Klebsiella pneumoniae, Salmonella, Shigella sonnei, Enterobacter spp, Kluyvera ascorbata, Citrobacter braakii, Raoultella ornithinolytica*) from the environment, food, humans, livestock, wildlife, companion animals, rivers, or vegetables in widespread geographical locations (Baron et al., 2016; Al-Tawfiq et al., 2017; Luo et al., 2017; Poirel et al., 2017).

Previous literatures have suggested that IncI2, IncX4, and IncHI2 are responsible for the worldwide distribution of *mcr-1* gene (Doumith et al., 2016; Cui et al., 2017; Li et al., 2017). IncI2, IncX4 and IncHI2 plasmids accounted for over 90% of reported *mcr-1*-bearing plasmids (Matamoros et al., 2017). However, mechanism of these three plasmid types being prevalent is still unclear, and the systematic evaluation regarding *mcr-1*-bearing plasmids carriage on its host fitness was scarce.

The *mcr-1* gene was first discovered in a pig *E. coli* strain SHP45 from a commercial farm where the prevalence of colistin resistance among *E. coli* isolates recovered from the pigs was over 50% (Liu et al., 2016). In this study, we aimed to characterize the molecular trait of *mcr-1*–positive plasmids among *Escherichia coli* strains from pigs of different ages in the farm where *mcr-1* was first identified and to evaluate the impact of diverse natural *mcr-1*–bearing plasmids on host fitness through *in vitro* competition assays.

## Materials and methods

### Sampling and bacterial isolates

Sixty rectal swab samples from pigs of different age groups (50 days old, 70 days old, 4 months old, and 5 months old) were collected from a pig farm located in Shanghai in July 2013, at which *mcr-1* was first reported. The antimicrobial application data at this farm over the past year were provided by the veterinarians (Table S1). The isolates were identified by conventional biochemical tests and confirmed by matrix-assisted laser desorption ionization–time of flight mass spectrometry (MALDI-TOF MS) (Shimadzu, Japan).

### Antimicrobial susceptibility test

The antimicrobial susceptibility test was performed using either the agar dilution method or broth microdilution method (limited to colistin), and the results were interpreted according to Clinical and Laboratory standards Institute (CLSI) recommendations (Clinical and Laboratory Standards Institute, 2017). The epidemiological cut-off value (http://mic.eucast.org/Eucast2/) for florfenicol and neomycin was >16 and > 8 mg/L, respectively. *E. coli* ATCC25922 was used as the reference strain.

### Detection of antimicrobial resistance genes

The strains were screened for the presence of *mcr-1* by PCR amplification using primers described previously (Table S2; Liu et al., 2016). Additionally, the *mcr-1*–positive isolates were also PCR-screened for the presence of the *bla*_CTX−M_, *fosA3, oqxAB*, and *floR* genes (Liu et al., 2007; Zhao et al., 2010; Hou et al., 2012; Li et al., 2015). PCR results were confirmed by sequencing.

### Molecular typing

Pulse-field gel electrophoresis (PFGE) was conducted to identify the clonal relationship among the *mcr-1*–positive isolates. The DNA of the isolates was digested with the restriction enzyme *Xba*I and then subjected to PFGE analysis using the CHEF-MAPPER System (Bio-Rad Laboratories, Hercules, CA, USA). The results were interpreted according to the criteria reported previously (Tenover et al., 1995).

### Conjugation and transformation experiments

Conjugation experiments were performed by the broth mating method with *E. coli* C600 (streptomycin resistant) used as the recipient strain. The transconjugants were selected on MacConkey agar plates supplemented with colistin (2 mg/L) and streptomycin (3,000 mg/L). Transfer frequencies were calculated as the ratio of transconjugants over recipient cells. Plasmids that failed to transfer by conjugation were extracted from the *mcr-1–*positive *E. coli* strains and transformed into the *E. coli* recipient strain DH5α (Takara) by the electroporation. Transformants were selected on Luria-Bertani agar plates containing 2 mg/L colistin. The antimicrobial susceptibility of the transconjugants/transformants was determined by either the agar dilution method or broth microdilution method, and the presence of *bla*_CTX−M_, *fosA3, oqxAB* and *floR* genes in the transconjugants/transformants was confirmed by PCR.

### Plasmid analysis

To determine the location of the *mcr-1* gene and the size of the plasmid, S1 nuclease pulsed-field gel electrophoresis (S1-PFGE) combined with Southern blotting was conducted. The samples were digested with the restriction enzyme *S*1 and separated using the CHEF-MAPPER System. The gels were run at 6.0 V/cm with an initial/final switch time of 2.16 s/63.8 s for 19 h. The plasmid DNA was transferred and cross-linked to positively charged nylon membranes (Roche Diagnostics) and hybridized using DIG-labeled *mcr-1* gene as the probe. PCR-based replicon typing (PBRT) was performed on all transconjugants/transformants, as described previously (Carattoli et al., 2005). Plasmid double locus sequence typing (pDLST) and replicon sequence typing (RST) were performed to better characterize the IncHI2 and IncFII plasmids, respectively (Garcia-Fernandez and Carattoli, 2010; Villa et al., 2010).

The genomic DNA of the transconjugants of SHP8, SHP10, SHP16, SHP23, SHP26, SHP41, SHP47, SHP48, SHP49, and SHP50 was extracted and sequenced using Illumina Hiseq 2000 (Illumina, San Diego, CA, USA). Sequence reads were assembled into contigs using SOAP denovo version 2.04. The contigs of 10 *mcr-1*–bearing plasmids, designated as pHNSHP8, pHNSHP10, pHNSHP16, pHNSHP23, pHNSHP26, pHNSHP41, pHNSHP47, pHNSHP48, pHNSHP49, and pHNSHP50, respectively, were separated from the chromosomal contigs and compared with our previously reported plasmids pHNSHP45 (Liu et al., 2016) and pHNSHP45-2 (Zhi et al., 2016) using Blast (http://blast.ncbi.nlm.nih.gov/Blast.cgi) and BRIG (Alikhan et al., 2011). Related *mcr-1*-carrying IncX4 plasmids were used to guide PCR-based gap closure, and Sanger sequencing was performed to assemble contigs of pHNSHP10, pHNSHP23, and pHNSHP49 into complete plasmids. The complete sequences of pHNSHP10, pHNSHP23, and pHNSHP49 and the partial sequences of the remaining seven plasmids were analyzed and annotated using RAST (Aziz et al., 2008), IS finder (https://www-is.biotoul.fr//), ResFinder (https://cge.cbs.dtu.dk//services/ResFinder/), and the Gene Construction Kit 4.0 (Textco BioSoftware, Inc., Raleigh, NC, USA).

### Genetic context of *mcr-1*

The surrounding regions flanking *mcr-1* and the insertion site of *mcr-1* in the plasmids were determined by PCR mapping and sequencing with primers listed in Table S3.

### Plasmid stability and growth kinetics

The stability of the *mcr-1*–positive plasmids was investigated *in vitro* according to a previously described protocol (Bryksin and Matsumura, 2010; Wang et al., 2017). In brief, transconjugants *E. coli* DH5α/pHNSHP45, *E. coli* DH5α/pHNSHP45-2, *E. coli* DH5α/pHNSHP23, *E. coli* DH5α/pHNSHP17, and *E. coli* DH5α/ pHNSHP24 were propagated by serial transfer for 14 days of passage. Periodically, the culture broths were serially diluted in 0.85% saline and plated onto LB agar without colistin. Approximately 100 colonies were randomly chosen and replica plated onto LB agar plates with colistin. ~50 colonies grown on colistin-supplemented agar were randomly selected to confirm the presence of *mcr-1* and the corresponding replicon type by PCR assay (Table S2; Carattoli et al., 2005). The growth kinetics of *E. coli* DH5α and its transformants carrying the plasmids pHNSHP23, pHNSHP45-2, pHNSHP45, pHNSHP17 and pHNSHP24 were studied by inoculation in 100 mL of fresh LB broth. The starting OD_600_ value was 0.01. Bacterial growth was recorded by monitoring OD_600_ every 1 h for 12 h at 37°C. Experiments were repeated in three separate assays.

### Competition experiments *in vitro*

To assess the fitness effect of *mcr-1-*bearing plasmids on bacterial host, *E. coli* plasmid-harboring transformants (*E. coli* DH5α with pHNSHP23, pHNSHP45-2, pHNSHP45, pHNSHP17 and pHNSHP24 representing IncX4, IncHI2, IncI2, IncY, and IncFII, respectively) was used to compete against plasmid-free *E. coli* DH5α. To further distinguish, plasmid-plasmid competition experiments were conducted between pair transformants (pHNSHP45/pHNSHP45-2, pHNSHP23/pHNSHP45-2, and pHNSHP45/pHNSHP23) in fresh LB as previously described (Foucault et al., 2009; Machuca et al., 2014). The overnight cultures of two comparable competitors were mixed at a rate of 1:1 at 0 h and 10^−3^ diluted into LB broth. The mixture was then incubated for 24 h, and then the mixed population was again 1000-fold diluted into fresh LB broth. This procedure was repeated until the competition experiment had lasted for 96 h. The total number of bacteria were determined by spreading properly diluted samples of each competition mixture on LB agar at 0, 24, 48, 72, and 96 h, and an average of 100 colonies were replica plated on LB agar supplemented with 2 mg/L colistin. The percentage of colistin resistant cells was deduced by counting the viable bacteria on colistin-supplemented LB agar, which were also analyzed by colony PCR targeting *mcr-1* gene and corresponding replicon type (Table S2; Carattoli et al., 2005).

The formula RF = (log_10_ S1_dt_ − log_10_ S1_d0_) / (log_10_ S2_dt_ − log_10_ S2_d0_) (Machuca et al., 2014) was used to calculate the relative fitness (RF), where S1 and S2 represent cfu densities of the constructed isolates/transformants and its control isolates (t is time in days). If there exists a fitness cost between the competitors, then RF < 1; if not, RF > 1. Statistical analysis was carried out via the software GraphPad Prism 5.0 (GraphPad Software Inc., La Jolla, CA).

### Nucleotide sequence accession numbers

The complete nucleotide sequences of pHNSHP10, pHNSHP23, and pHNSHP49, and the partial sequences of *mcr-1*-bearing plasmids pHNSHP8, pHNSHP16, pHNSHP26, pHNSHP47, pHNSHP48, and pHNSHP50 were deposited in GenBank under the accession numbers MF774182, MF774184, MF774188, MF774181, MF774183, MF774185, MF774186, MF774187, and MF774189, respectively.

## Results

### Incidence of *mcr-1*

Forty-seven *E. coli* isolates were recovered from the 60 samples obtained from pigs of different ages in the farm. Among them, 27 (57.5%) showed resistance to colistin (>2 mg/L) and were positive for *mcr-1* (Table 1).

**Table 1:** Details of the *mcr-1* positive *E. coli* isolates from swine.

**Isolates**	**Age group**	**PFGE pattern**	**MIC of colistin (mg/L)**	**Other resistance phenotype^c^**	**Genetic environment**	**Replicon**	**Conjugation frequency**	**Plasmid size (kb)**	**Other resistance gene (s)**
SHP50	50 days	F	4	FFC, TET, CIP, SXT	IncI2-*mcr-1*-IncI2	IncI2	4 × 10^−4^	~60	*oqxAB, floR*
SHP52	50 days	G	4	AMP, FFC, NEO, TET, CIP	IncX4-*mcr-1*-IncX4	IncX4	1.27 × 10^−3^	~33	*oqxAB, floR*
SHP49, SHP36	50 days	E	8	AMP, FFC, GEN, NEO, CIP, SXT	IncX4-*mcr-1*-IncX4	IncX4	2 × 10^−5^, 1.78 × 10^−4^	~33	*oqxAB, floR*
SHP47	50 days	P	8	*AMP, CTX, FFC, FOS, GEN, NEO, SXT*	IS*ApI1*-*mcr-1*-IncHI2	IncHI2 (ST3)	2 × 10^−6^	~244	*bla_*CTX*−*M*−14_*, *floR*, *fosA3*, *oqxAB*
SHP48	50 days	Q	8	*NEO, FFC, SXT, TET, CIP*	IS*ApI1*-*mcr-1*- IncHI2	IncHI2 (ST3)	–	~275	*oqxAB, floR*
SHP39	50 days	N	4	*AMP, CTX, FFC, FOS, GEN, NEO, TET, SXT*	IS*ApI1*-*mcr-1*-IncHI2	IncHI2 (ST3)	2 × 10^−6^	~244	*bla_*CTX*−*M*−14_*, *floR*, *fosA3*, *oqxAB*
SHP41	50 days	O	4	*AMP, CTX, FFC, FOS, TET, SXT*	IS*ApI1*-*mcr-1*- IS*ApI1*, IncX4-*mcr-1*-IncX4	IncHI2 (ST3), IncX4	–	~238, ~33	*bla_*CTX*−*M*−15_*, *floR*, *fosA3*, *oqxAB*
SHP51	50 days	R	8	*AMP, CTX, FFC, FOS, GEN, NEO, TET, SXT*	IS*ApI1*-*mcr-1*-IncHI2, IS*ApI1*-*mcr-1*- IS*ApI1*	IncHI2 (ST3)	2.90 × 10^−5^	~244, chromosome	*bla_*CTX*−*M*−14_*, *floR*, *fosA3*
SHP43	50 days	D3	8	*AMP, CTX, FFC, FOS, GEN, NEO, CIP, SXT*	IS*ApI1*-*mcr-1*-IncHI2	IncY-IncHI2	–	~350	*bla_*CTX*−*M*−14_*, *floR*, *fosA3*, *oqxAB*
SHP45	50 days	D2	8	*AMP, CTX, FFC, FOS, GEN, NEO, TET, CIP, SXT*	IS*ApI1*-*mcr-1*-IncI2, IS*ApI1*-*mcr-1*-IncHI2	IncI2, IncHI2 (ST3)	–	~64, ~244	*bla_*CTX*−*M*−14_*, *floR*, *fosA3*, *oqxAB*
SHP26	70 days	D1	8	*AMP, CTX, FFC, FOS, GEN, NEO, TET, CIP, SXT*	IS*ApI1*-*mcr-1*-IncHI2	IncHI2 (ST3)	6.84 × 10^−5^	~244	*bla_*CTX*−*M*−14_*, *floR*, *fosA3*, *oqxAB*
SHP46^a^	50 days	K2	4	AMP, FFC, NEO, SXT	IS*ApI1*-*mcr-1*-?	ND	–	~104	*oqxAB, floR*
SHP22	70 days	K1	8	AMP, FFC, NEO, SXT	IS*ApI1*-*mcr-1*- IS*ApI1*	F29:A-:B-	–	~78	*floR*
SHP17	70 days	J	4	AMP, CTX, FFC, NEO, TET, SXT	unknown	IncY	8.64 × 10^−6^	~83	*bla*_CTX−M−55_, *oqxAB, floR*
SHP28	70 days	J	8	AMP, CTX, FFC, NEO, TET, SXT	IncX4-*mcr-1*-IncX4	IncX4	–	~33	*oqxAB, floR*
SHP31^a^	70 days	L	8	FFC, NEO, TET, CIP, SXT	ISApI1*-mcr-1*- IS*ApI1*	ND	–	~80	*oqxAB, floR*
SHP24	70 days	L	8	FFC, NEO, TET, SXT	IS*ApI1*-*mcr-1*- IS*ApI1*	F53:A-:B-	–	~80	*floR*
SHP33^a^	70 days	M	16	FFC, NEO, TET, CIP, SXT	unknown	NA	–	chromosome	*oqxAB, floR*
SHP23	70 days	smeared	8	AMP, FFC, NEO, TET, CIP, SXT	IncX4-*mcr-1*-IncX4	IncX4	5.43 × 10^−5^	~33	*oqxAB, floR*
SHP32	70 days	A	4	AMP, FFC, TET, CIP, SXT	IncX4-*mcr-1*-IncX4	IncX4	–	~33	*oqxAB, floR*
SHP14^b^	4 months	A	8	AMP, FFC, GEN, TET, CIP, SXT	IncX4-*mcr-1*-IncX4	IncX4	–	~33	*oqxAB, floR*
SHP16	4 months	I	8	TET, SXT	IS*ApI1*-*mcr-1*-IncI2	IncI2	8.12 × 10^−5^	~64	*oqxAB*
SHP10	4 months	H	8	AMP, FFC, NEO, TET, SXT	IncX4-*mcr-1*-IncX4	IncX4	5 × 10^−6^	~33	*oqxAB, floR*
SHP8	4 months	C	8	AMP, FFC, NEO, TET, SXT	IS*ApI1*-*mcr-1*-IncI2	IncI2	–	~62	*oqxAB, floR*
SHP7	4 months	B	4	AMP, CTX, FOX, FFC, TET, SXT	IncX4-*mcr-1*-IncX4, IS*ApI1*-*mcr-1*-IncI2	IncX4, IncI2	–	~33, ~62	*oqxAB, floR, bla*_CMY−2_
SHP59	5 months	S	4	AMP, FFC, TET, SXT	IncX4-*mcr-1*-IncX4	IncX4	–	~33	*oqxAB, floR*

a *Isolates failed to get transconjugants*.

b *Isolates successfully get transformant by the electroporation*.

c *AMP, ampicillin; CIP, ciprofloxacin; CTX, cefotaxime; FFC, florfenicol; FOS, fosfomycin; GEN, gentamicin; NEO, neomycin; SXT, sulfamethoxazole-trimethoprim; TET, tetracycline. Patterns transferred by conjugation are underlined*.

### Characterization of *mcr-1*–positive *E. coli* isolates

PFGE was successfully performed in 26 of the 27 *mcr-1*–positive *E. coli* isolates, which were grouped into 19 clonal patterns, designated A to S (Table 1 and Table S1). This result suggested that the dissemination of the *mcr-1* gene in this farm was not mainly due to clonal expansion. The *mcr-1*–positive *E. coli* isolates showed MICs of 4–16 mg/L for colistin. All of the *mcr-1* producers displayed the multidrug resistance phenotype (Table 1).

### Plasmid analysis and location of the *mcr-1* genes

S1-PFGE and Southern hybridization analysis confirmed that the *mcr-1* genes in the 26 *E. coli* isolates were located on plasmids ranging in size from 33 to 350 kb, whereas the *mcr-1* gene in strain SHP33 was chromosomally located (Table 1). Two *mcr-1*–bearing plasmids coexisted in three isolates (SHP45, SHP41, and SHP7). In addition, two copies of the *mcr-1* genes, one located on a chromosome and the other on a ~244-kb plasmid, were detected in strain SHP51. The *mcr-1–*bearing plasmids of the 23 *E. coli* isolates were successfully transferred to recipients by conjugation, and one transformant carrying *mcr-1* positive plasmid was obtained by transformation. In seven transconjugants, ampicillin, cefotaxime, florfenicol, and fosfomycin resistance and reduced susceptibility to ciprofloxacin were co-transferred when compared to that in the parent recipient strain, which was due to the co-transfer of *bla*_CTX−M−14_, *fosA3, floR*, and *oqxAB* genes (Table 1). The IncI2, IncX4, and IncHI2/ST3 plasmids were detected in 5, 10, and 7 transconjugants, respectively. The other 4 *mcr-1*–bearing plasmids included one hybrid plasmid containing both IncY and IncHI2 replicons, one IncY plasmid, and two IncFII plasmids classified as F29:A-:B- and F53:A-:B- by replicon sequence typing (RST). Interestingly, all of the *mcr-1*–bearing IncHI2 plasmids were detected in isolates from nursery piglets (50–70 days old). The conjugation frequency varied from 10^−2^ to 10^−6^ transconjugants per recipient.

Given the large number of *mcr-1*–bearing IncI2, IncX4, and IncHI2 plasmids in this farm, ten plasmids comprising three IncI2-, three IncX4-, and four IncHI2-type plasmids were randomly chosen as representatives of each plasmid type to be sequenced. Among them, the complete sequence was obtained for the three IncX4 plasmids pHNSHP49, pHNSHP23 and pHNSHP10. These plasmids are highly similar to other *mcr-1*–bearing IncX4 plasmids such as pAF48 (KX032520; *E. coli*) in Switzerland, pESTMCR (KU743383; *E. coli*) from Estonia, pICBEC72Hmcr (CP015977; *E. coli*) in Brazil, pMCR1-NJ-IncX4 (KX447768; *E. coli*) in the United States, and pMCR1_Incx4 (KU761327; *Klebsiella pneumoniae*) from China. They all have a typical IncX4 backbone including the identical replication initiation protein gene *pir*, conjugal transfer protein gene *trbM, taxABC*, and *pilX* operons. The upstream insertion sequence IS*Apl1* flanking *mcr-1* was consistently absent, and a previously described *pap2* gene and the insertion sequence IS*26* were present around *mcr-1*. No resistance genes other than *mcr-1* were found (Figure S1a).

The remaining seven plasmids were also compared with other corresponding plasmids from various sources all over the world, using plasmid-related contigs. The IncI2-type plasmids, namely pHNSHP8, pHNSHP16, and pHNSHP50, showed 99% coverage to our previously reported plasmid pHNSHP45 (KP347127), the first identified *mcr-1*–bearing plasmid obtained from the same farm in 2013 (Liu et al., 2016). These three plasmids were also similar to other IncI2 plasmids such as pA31-12 (KX034083; *E. coli*; chicken), pZE36 (KY802014; *E. coli*; human), pEc_27COE18 (KY012275; *E. coli*; human), pECJS-61-63 (KX254342; *E. coli*; swine) from China, pMRY15-117_2DNA (AP017619; *E. coli*; unknown) from Japan, pOM97-mcr (KY693674; *E. coli*; human) from Oman, and pSLy21 (NZ_CP016405; *E. coli*; unknown) from the United States, all of which harbored a typical IncI2 backbone including a replicon region, plasmid stability function region, and conjugative transfer regions (*tra* and *pil* genes) (Figure S1b). The *tnpA*-IS*683* region present in plasmid pHNSHP45 was also present in pHNSHP8 with the same insertion site and orientation, but was absent in other plasmids, including pHNSHP16 and pHNSHP50 in our study. As for IncHI2 plasmids pHNSHP26, pHNSHP41, pHNSHP47, pHNSHP48, they possessed most of the IncHI2 plasmid backbone sequences and were similar to each other, as well as to the previously reported plasmid pHNSHP45-2 (KU341381) (Zhi et al., 2016) with the same *mcr-1*-IS*Apl1* insertion sites and orientation, except that *mcr-1* was flanked by two copies of IS*Apl1* with different insertion site on plasmid pHNSHP41. A slight difference was observed in the MDR regions of these plasmids, and part of the conjugative transfer regions (*htdZKLVT* and *trhBCELKV*) was absent in pHNSHP48 (Figure S1c).

### Genetic background of *mcr-1*

PCR mapping and sequencing revealed that the genetic context of *mcr-1* was diverse. Among the 10 plasmids, including 6 IncHI2 and 4 IncI2 plasmids, a single copy of IS*Apl1* was found to be located upstream of *mcr-1*. On 5 plasmids, the *mcr-1* gene was flanked by two copies of IS*Apl1*. In 10 plasmids, including all IncX4 plasmids, no copy of IS*Apl1* was present. In addition, using primers located on the IncX4, IncI2, or IncHI2 backbone and the *mcr-1* gene, we confirmed that *mcr-1* was inserted at the same sites on the IncX4, IncI2, and IncHI2 plasmids as that on plasmids pHNSHP23, pHNSHP45, and pHNSHP45-2, respectively, which indicated that pHNSHP23-like, pHNSHP45-like, and pHNSHP45-2-like plasmids were prevalent in this pig farm.

### Plasmid stability and growth curve

**Figure 1:**
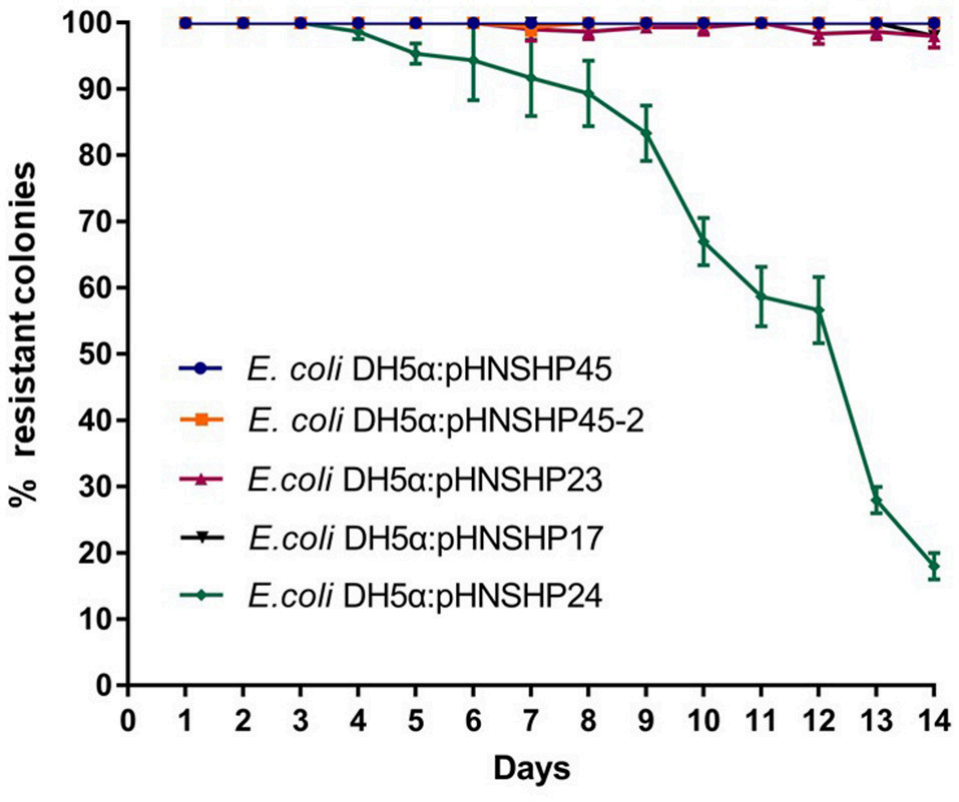
*In vitro* stability of plasmids pHNSHP23, pHNSHP45-2, pHNSHP45, pHNSHP17 and pHNSHP24, data shown are the means of three independent assays. The error bars denote the 95% CI.

**Figure 2:**
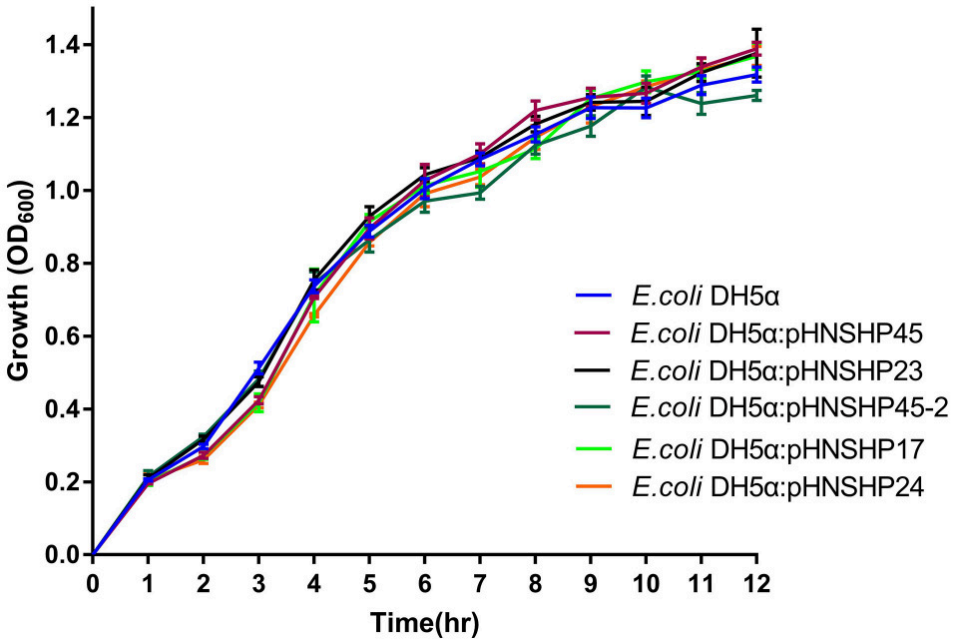
Growth curve of *E. coli* DH5α and isogenic transformants. Curve indicates the mean of three independent experiments. The error bars denote the 95% CI.

The stability of plasmids pHNSHP45(IncI2), pHNSHP45-2(IncHI2), pHNSHP23(IncX4), pHNSHP17(IncY), and pHNSHP24(IncFII) was determined. No plasmid loss was detected during 96 h of competition experiments. pHNSHP45, pHNSHP45-2, pHNSHP23, and pHNSHP17 maintains stable in *E. coli* for at least 14 days of passage in an antibiotic-free environment. However, plasmid loss occurred in transformants *E. coli* DH5α/pHNSHP24 from day 5 (Figure 1). Growth kinetics of the transformants with these six plasmids and *E. coli* DH5α were also investigated. No obvious difference in growth was observed between the transformants and the recipient strain after 12 h assessment (Figure 2).

### Fitness effects of *mcr-1* gene and *mcr-1* plasmids *in vitro*

**Figure 3:**
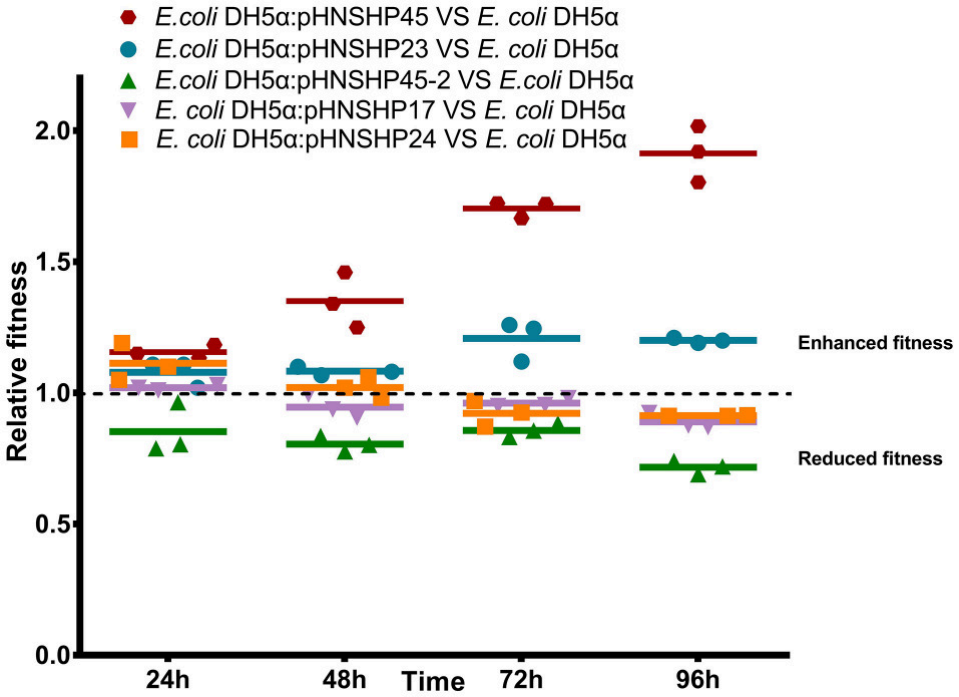
Relative fitness of transformants with *mcr-1*–bearing plasmids pHNSHP23, pHNSHP45-2, pHNSHP45, pHNSHP17, and pHNSHP24 *in vitro* competition.

**Figure 4:**
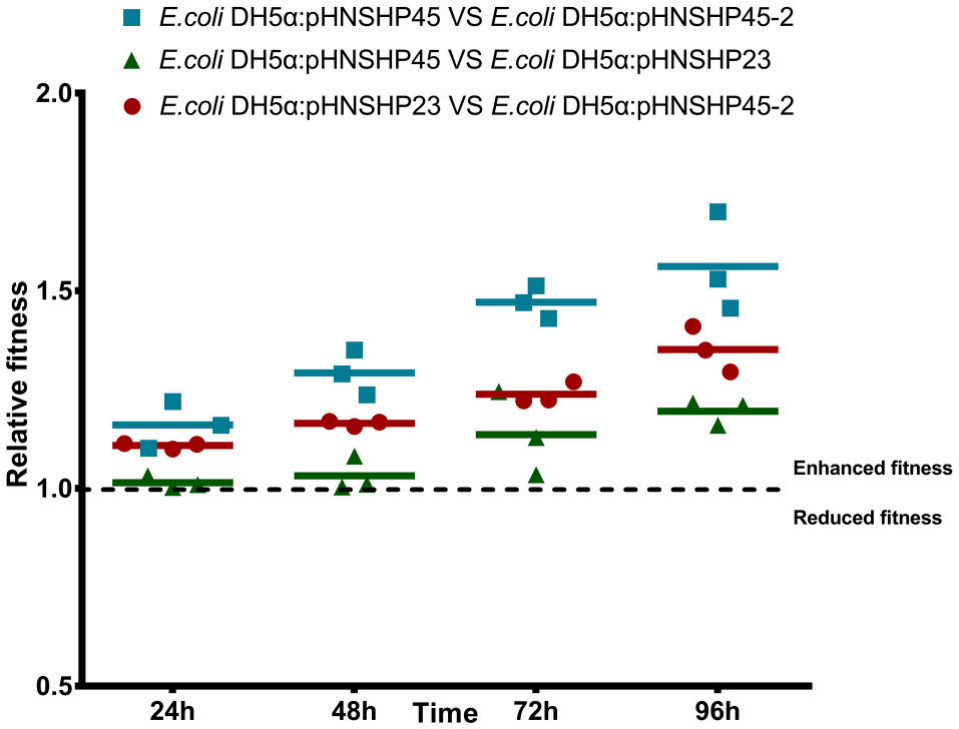
Growth competition *in vitro* between strains transformed with plasmids pHNSHP45 and pHNSHP45-2, pHNSHP23 and pHNSHP45-2 as well as pHNSHP45 and pHNSHP23.

The impact of plasmids pHNSHP45, pHNSHP45-2, pHNSHP23, pHNSHP17 and pHNSHP24 on host fitness was evaluated by direct competition assays of plasmid-carrying *E. coli* DH5α against plasmid-free *E. coli* DH5α. The transformants with pHNSHP45(IncI2) or pHNSHP23(IncX4) outnumbered the parent *E. coli* DH5α strain after 24, 48, 72, and 96 h of cultivation. Plasmid pHNSHP45 in particular presented a high fitness advantage for its host, and the fitness was enhanced (16–91%) as serial passage. In contrast, plasmid pHNSHP45-2(IncHI2) imposed a slight fitness cost from 15% at 24 h to 28% at 96 h. For transformants *E. coli* DH5α/pHNSHP17 and *E. coli* DH5α/pHNSHP24, a slight fitness increase (2% and 11%, respectively) was observed at 24 h of passage, but the fitness was vanished following continuous growth, *E. coli* DH5α outcompete DH5α/pHNSHP17 or *E. coli* DH5α/pHNSHP24 after 48 or 72 h (Figure 3). Considering the fitness advantage of pHNSHP45 and pHNSHP23 described above and epidemiological success of IncI2, IncHI2 and IncX4 plasmids that *mcr-1* located, we further compared the fitness of transformants with these three types of plasmids in a co-growth experiment, the outcome of plasmid-plasmid competition assays showed that under free-antimicrobials environment, *E. coli* DH5α acquiring pHNSHP45 outcompeted transformants with pHNSHP23 with a related fitness rate of 1.01–1.21 monitored from 24 to 96 h. While similar to the result mentioned above, pHNSHP45-2 plasmid was relative to a significant competition disadvantage vs. pHNSHP23 or pHNSHP45 (Figure 4).

## Discussion

Previous literatures have suggested that multiple mobile elements especially plasmids contributed to the rapid spreading of *mcr-1* (Li et al., 2017; Matamoros et al., 2017). Therefore, we characterized *mcr-1*-positive plasmids in this pig farm. The phnshp23-like (incx4), phnshp45-like (inci2), and phnshp45-2-like (inchi2) were the most common plasmids in the investigated strains, suggesting their dissemination was primarily responsible for the high rate of *mcr-1* among *e. Coli* isolates in the pig farm. It is consistent with previous reports that the worldwide distribution of *mcr-1* gene has mainly mediated by incx4, inci2, and inchi2-type plasmids (Falgenhauer et al., 2016; Poirel et al., 2016, 2017; Veldman et al., 2016; Li et al., 2017; Liu et al., 2017; Matamoros et al., 2017; Roschanski et al., 2017), with inci2 being the most prevalent plasmid backbone, followed by incx4 and inchi2. Thus, we further compared *mcr-1*–bearing incx4, inci2, and inchi2 plasmid content deposited in genbank, and found that *mcr-1*–bearing incx4 and inci2 plasmids detected in different countries were similar to the respective plasmids in our study, including the plasmid backbone and the *mcr-1* insertion site. Although the insertion site of *mcr-1* in inchi2 varied, almost all of the *mcr-1*–bearing inchi2 plasmids belonged to st3, especially those from china (Liu et al., 2017; Luo et al., 2017). In addition, all inchi2 plasmids that carried the *bla*_ctx−m_*, fosa3, flor*, and *mcr-1* genes were identified among the isolates from nursery pigs. Considering the nursery pigs had been treated or fed with several antimicrobials, it seemed that the high selective pressure posed by the use of antimicrobials contributed to the spread of multidrug resistant inchi2 plasmids in the nursery pigs. However, because of the limitation in sample amount collected in each age group, this association still need to be further determined.

It is commonly considered that the variable fitness caused by plasmids depends on different host-plasmid combinations (Bouma and Lenski, 1988). Previous studies described that *mcr-1*-bearing plasmid can incur fitness cost on the bacterial host, but the fitness cost was caused by a IncHI2-type plasmid carrying *mcr-1* (He et al., 2017). Thus, we systematically evaluated the fitness of a series of natural epidemic plasmids on the bacteria, and found that *mcr-1*–bearing IncI2, IncX4, and IncHI2 plasmids were quite stable and would not affect the growth of their hosts, which explained why these plasmids were dominant in this pig farm and worldwide. The result is in consistence with the previous reports that carriage of pGZ2-mcr (IncI2 type plasmid) or pECMCR-1101 (IncX4 type plasmid) would not impair the growth of recipient *E. coli* J53 or C600 (Kong et al., 2017; Zhang et al., 2017). Additionally, the fitness advantage of IncI2 and IncX4 plasmids on the host strain as well as the competitive advantage of IncI2 and IncX4 plasmids against IncHI2 plasmids (IncI2 plasmid type possess strongest competitive capability) further explained the fact that most reported *mcr-1*-bearing plasmids primarily belong to IncI2 and IncX4 plasmid types, especially IncI2 plasmids (Li et al., 2017; Matamoros et al., 2017) and their persistence in fattening pigs without the antibiotic exposure. Whereas the fitness cost imposed by IncHI2 plasmid type could give a clue to the less prevalence of IncHI2 plasmid type comparing to IncI2 and IncX4 and its missing in fattening pigs. Thus, IncI2 as well as IncX4 plasmids, due to their enhanced fitness of hosts, competition advantage over other plasmid types and plasmid stability, are able to replace other plasmids becoming capital vehicles for *mcr-1* dissemination. Furthermore, a recent study has confirmed that the substantially increased expression of *mcr-1* will impose a fitness burden on its *E. coli* host (Yang et al., 2017). The moderate MICs of colistin, primarily maintained among 4-8 mg/L in this study, and the fitness benefit mediated by *mcr-1*-bearing IncI2 and IncX4 plasmids as well as their epidemiological success further supported the notion that natural bacteria tend to keep a balance between the capability of resistance to colistin and its fitness impact on its host (Yang et al., 2017).

In conclusion, IncI2, IncX4, and IncHI2 were the major plasmid types driving the dissemination of *mcr-1* in this farm. Plasmid stability and increased fitness or co-selection by other antimicrobials might contribute to the dissemination of the three epidemic *mcr-1*–positive plasmids (IncI2, IncX4, and IncHI2) in this farm and worldwide. Though China has now stopped colistin as a growth promoter (Walsh and Wu, 2016), the combination of multi-drug resistance capability of IncHI2-type plasmids and the fitness advantage conferred by the IncX4 and IncI2 plasmids indicated their potential for further dissemination with or without antibiotic selection pressure. We suggested that beside colistin, it is also essential to conduct prudent management for overall antimicrobials.

## Ethics statement

This study was carried out in accordance with the recommendation of ethical guidelines of South China Agricultural University. Individual written informed consent for the use of fecal samples was obtained from all animal owners.

## Author contributions

J-HL designed the study. RW, LFY, LXY, YL, and XC carried out the experiments. RW, LXY, JW, LL, and JY analyzed the data. LXY and J-HL wrote the manuscript. J-HL and JW revised the manuscript. All authors read and approved the final manuscript.

### Conflict of interest statement

The authors declare that the research was conducted in the absence of any commercial or financial relationships that could be construed as a potential conflict of interest.
