# Supplementary material for: Fitness Advantage of mcr-1–Bearing IncI2 and IncX4 Plasmids in Vitro
Source: Front Microbiol. 2018 Feb 27;9:331. doi: 10.3389/fmicb.2018.00331 (PMC5835064; doi:10.3389/fmicb.2018.00331)
Supplement: Supplementary file 1 [file DataSheet1.DOC]

**Supplementary material**

**Table S1. Prevalence of *mcr-1*-carrying *E. coli* isolates from pigs of different ages and information on antimicrobial use.**

| Stage | Number of isolates | Positive isolates  (Percentage) | PFGE pattern of *mcr-1*- positive isolates | Antimicrobials used  for treatment | | Feed additives |
| --- | --- | --- | --- | --- | --- | --- |
| 50 days | 14 | 12 (85.7%) | 10 | ceftiofur, amoxicilin, florfenicol | colistin, olaquindox, Nosiheptide, copper sulfate, manganese sulfate, zinc oxide | |
| 70 days | 11 | 9 (81.8%) | 6 (one smeared) | ceftiofur, amoxicilin, florfenicol | colistin, olaquindox, Nosiheptide, copper sulfate, manganese sulfate, zinc oxide | |
| 4 months | 13 | 5 (38.5%) | 5 | no | copper sulfate, manganese sulfate | |
| 5 months | 9 | 1 (11.1%) | 1 | no | copper sulfate, manganese sulfate | |
| Total | 47 | 27 (57.5%) | 19(one smeared) |  |  | |

**Table S2. Primers used for PCR and DNA sequencing in this study**

| Gene | Primer name | Sequence (5’ to 3’) | Reference |
| --- | --- | --- | --- |
| *mcr-1* | MCR1-F | CGGTCAGTCCGTTTGTTC | 1 |
|  | MCR1-R | CTTGGTCGGTCTGTAGGG | 1 |
| *fosA3* | FosA3-F | GCGTCAAGCCTGGCATTT | 2 |
|  | FosA3-R | GCCGTCAGGGTCGAGAAA | 2 |
| *floR* | FloR-F | GCGATATTCATTACTTTGGC | 3 |
|  | FloR-R | TAGGATGAAGGTGAGGAATG | 3 |
| *oqxAB* | oqxAB-F | GTCCAGCGATAATCAGGC | 4 |
|  | oqxAB-R | GGTCTCGGCAATCACTTT | 4 |
| *bla*_CTX-M-1_ | bla_CTX-M-1_-F | CTTCCAGAATAAGGAATCCC | 5 |
|  | bla_CTX-M-1_-R | CGTCTAAGGCGATAAACAAA | 5 |
| *bla*_CTX-M-9_ | bla_CTX-M-9_-F | TGACCGTATTGGGAGTTTG | 5 |
|  | bla_CTX-M-9_-R | ACCAGTTACAGCCCTTCG | 5 |

**Table S3.** Primers used for determining genetic environment of *mcr-1*

| Primer | Sequence (5’-3’) | Position | Corresponding genetic context | Size (bp) | Annealing  Temp. (℃) | Reference |
| --- | --- | --- | --- | --- | --- | --- |
| Mhp-F | TTGCCAGATTTGCTACTGT | downstream of *mcr-1* | *mcr-1*-IS*Apl1* | 696 | 54 | This study |
| ISAp-R | TTTCTCGCTCGTTTATTGTA | IS*Apl1* |  |  |  |  |
| Mcrd-F | GCTGATCTACTTGCTGGTTG | downstream of *mcr-1* | *mcr-1*-IncHI2 | 832 | 53.5 | This study |
| HI2-R | ATGGATGATTTCGGTTCG | IncHI2 backbone |  |  |  |  |
| ISAP-F | CGAAGCACCAAGACATCA | IS*ApI1* | IS*Apl1- mcr-1* | 393 | 55 | 6 |
| MCR-R | CCACAAGAACAAACGGACT | *mcr-1* |  |  |  |  |
| IncI2-F | AGTGGATGTTACGGAGCAG | IncI2 backbone | IncI2-mcr-1 | 894 | 57 | This study |
| mprA-R | CCACAAGAACAAACGGACT | *mcr-1* |  |  |  |  |
| clrAD-F | GTATCTGGTGCTGACTTTGA | downstream of *mcr-1* | *mcr-1-*IncI2 | 723 | 53.5 | This study |
| IncI2-R | ACTTAGCGATCTCGTTGTT | IncI2 backbone |  |  |  |  |
| IncX4-F3 | AGAGCTTGAGGGAATAGAA | IncX4 backbone | IncX4-*mcr-1* | 879 | 53 | This study |
| Mcr-R3 | CACAGGCTTTAGCACATAG | *mcr-1* |  |  |  |  |
| Mcr-F5 | AACGGTGTCTATCTACATGGTAT | *mcr-1* | *mcr-1-* IncX4 | 1674 | 53 | This study |
| IncX4-R5 | CATTGAATTTGTTCGTCCTC | IncX4 backbone |  |  |  |  |

**References**

1. Liu, Y.Y., Wang, Y., Walsh, T.R., Yi, L.X., Zhang, R., and Spencer, J., et al. (2016). Emergence of plasmid-mediated colistin resistance mechanism MCR-1 in animals and human beings in China: a microbiological and molecular biological study. *Lancet Infect. Dis.* 16, 161-8. doi: 10.1016/S1473-3099(15)00424-7.
2. Hou, J., Huang, X., Deng, Y., He, L., Yang, T., and Zeng, Z., et al. (2012). Dissemination of the fosfomycin resistance gene *fosA3* with CTX-M beta-lactamase genes and *rmtB* carried on IncFII plasmids among *Escherichia coli* isolates from pets in China. Antimicrob. Agents Chemother. 56, 2135-8. doi: 10.1128/AAC.05104-11.
3. Li, B., Zhang, Y., Wei, J., Shao, D., Liu, K., and Shi, Y., et al. (2015). Characterization of a novel small plasmid carrying the florfenicol resistance gene *floR* in *Haemophilus parasuis*. J. Antimicrob. Chemother.70, 3159-61. doi: 10.1093/jac/dkv230.
4. Zhao, J., Chen, Z., Chen, S., Deng, Y., Liu, Y., and Tian, W., et al. (2010). Prevalence and dissemination of *oqxAB* in

*Escherichia coli* isolates from animals, farmworkers, and the environment. Antimicrob. Agents Chemother. 54, 4219-24. doi: 10.1128/AAC.00139-10.

1. Liu, J.H., Wei, S.Y., Ma, J.Y., Zeng, Z.L., Lu, D.H., and Yang, G.X., et al. (2007). Detection and characterisation of CTX-M and CMY-2 beta-lactamases among *Escherichia coli* isolates from farm animals in Guangdong Province of China. Int. J. Antimicrob. Agents 29, 576-81. doi: 10.1016/j.ijantimicag.2006.12.015.

**6.** Yi, L., Wang, J., Gao, Y., Liu, Y., Doi, Y., and Wu, R., et al. (2017). mcr-1-Harboring Salmonella enterica Serovar Typhimurium Sequence Type 34 in Pigs, China. Emerg. Infect. Dis. 23, 291-295. doi: 10.3201/eid2302.161543.

**Figure S1.** Structural comparison of IncX4 (a), IncI2 (b), and IncHI2 (c) type plasmids. (a) The outer circle in black with annotation is the reference plasmid. (b) The circle in red with annotation indicates the reference plasmids. (c) The reference plasmid is annotated with red arrows. Information about plasmids appearing in the chart has been illuminated in text.


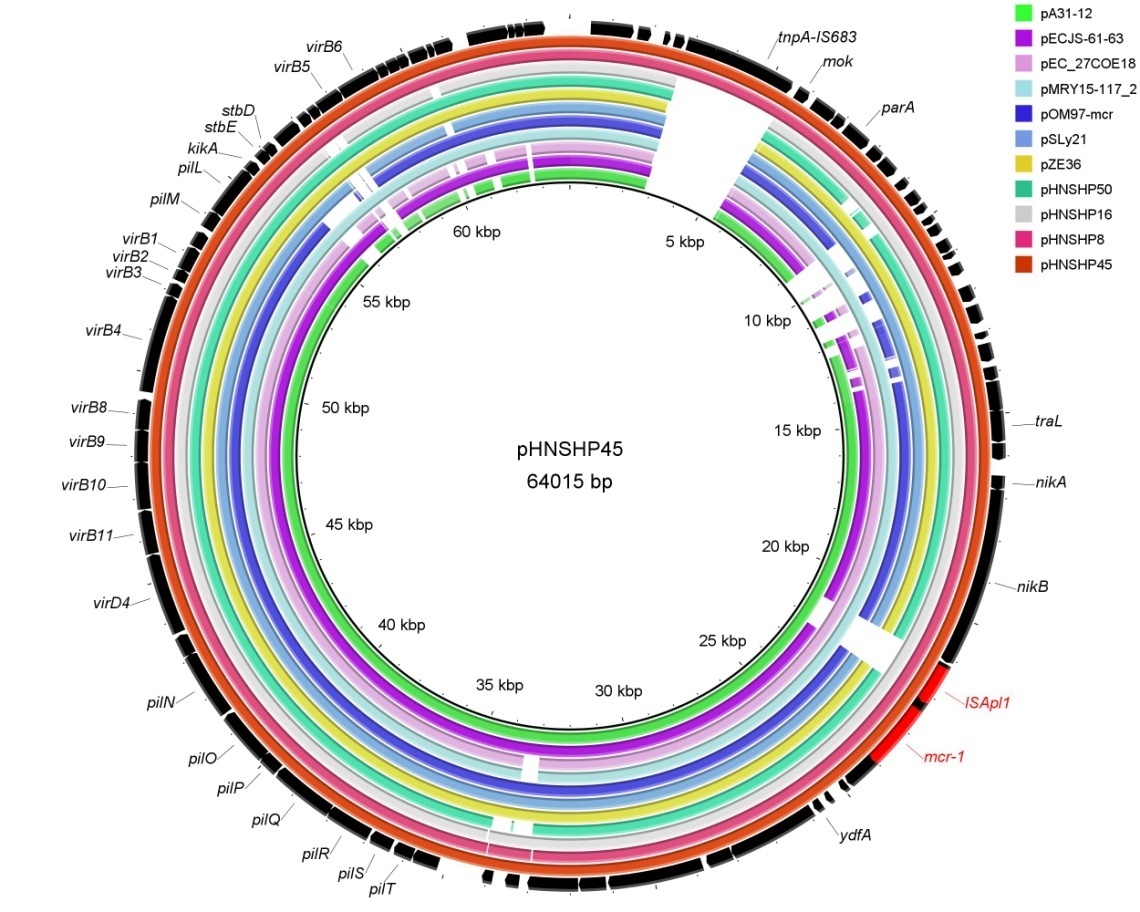


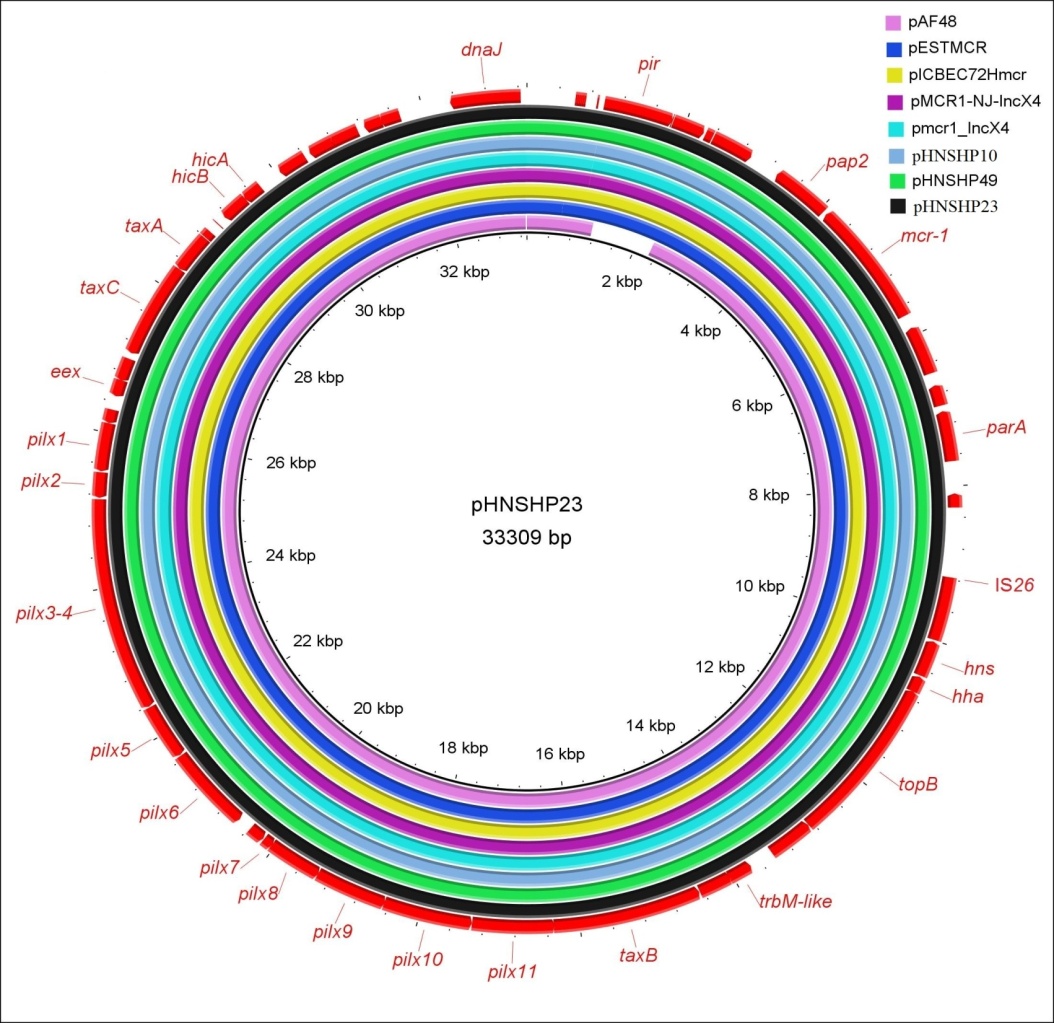


a.

b.

c.

**
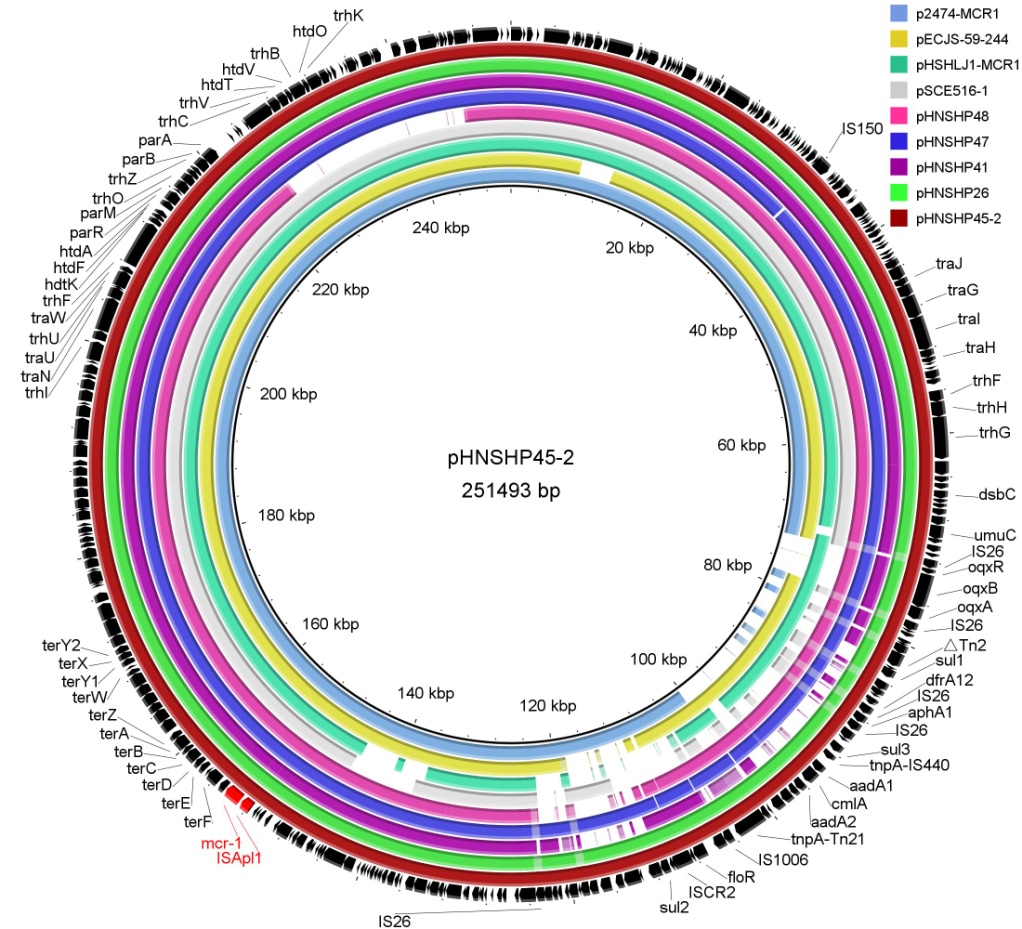
**
